# A community based participatory research approach to evaluate barriers and facilitators for behavioral weight loss intervention implementation in a predominantly black community

**DOI:** 10.1007/s00520-026-10548-7

**Published:** 2026-04-06

**Authors:** Elizabeth De Jesus, Sean Woo, Robert S. Krouse, Carmen E. Guerra, Katharine A. Rendle, Tamara Cadet, Kelly C. Allison, Julia Tchou

**Affiliations:** 1 University of Pennsylvania, Philadelphia, PA USA; 2 Department of Biostatistics, Epidemiology and Informatics, University of Pennsylvania, Philadelphia, PA USA; 3 Perelman School of Medicine, University of Pennsylvania, Philadelphia, PA USA; 4 Department of Family Medicine & Community Health, Perelman School of Medicine, University of Pennsylvania, Philadelphia, PA USA; 5 School of Social Policy & Practice, University of Pennsylvania, Philadelphia, PA USA; 6 Department of Psychiatry, Perelman School of Medicine, University of Pennsylvania, Philadelphia, PA USA; 7 Abramson Cancer Center, University of Pennsylvania, Philadelphia, PA USA

**Keywords:** Breast cancer, Weight loss, Obesity, Healthcare disparity

## Abstract

**Purpose:**

Breast cancer survivors with overweight or obesity have worse breast cancer specific survival and outcomes as compared to those with average weight by Yung and Ligibel (Clin Adv Hematol Oncol 14:790–797, 2016) and Druesne-Pecollo et al. (Breast Cancer Res Treat 135:647–654, 2012). Our pilot Group-bas*E*d *T*elehealth behavioral *We*ight *L*os (GET-WEL) Program showed that fewer Black breast cancer survivors (BBCS) enrolled and lost less weight than White breast cancer survivors by Allison et al. (Obesity Science and Practice 10:e70023, 2024). This study is aimed at using a community-based participatory research approach to assess barriers and facilitators of implementing a behavioral weight loss intervention among BBCS.

**Methods:**

Eight BBCS from a predominantly Black community were invited to participate in semi-structured interviews that were voice recorded, transcribed, coded, and analyzed via comparative thematic analysis.

**Results:**

Thematic analyses revealed multiple barriers within participants. These included lack of affordable healthy food access, safety concerns with regards to outdoor activities, lack of affordable fitness center memberships, time constraints related to competing work/life obligations, and steep learning curves with technology use. Most BBCS preferred an integrated community-based coach to guide their weight loss interventions via a combination of both virtual and in-person sessions.

**Conclusion:**

Our results indicate that a multimodal approach including nutrition education, reducing physical activity barriers, limiting time constraints by implementing both in-person and virtual platforms, and assisting with technology courses, is necessary to improve the equitable implementation of weight loss interventions. BBCS recommended utilizing established community facilities and leveraging known community members such as nutrition counselors and physical trainers to increase successful implementation.

## Introduction

Breast cancer survivors with body mass indexes (BMI) that are categorized with overweight [BMI 25–29.9 kg/m^2^] or obesity (BMI > 30) have worse breast cancer specific survival and outcomes as compared to breast cancer survivors with normal BMI (BMI of 18.5–24.9 kg/m^2^) [1, 2]. Worse breast cancer-specific survival includes higher recurrence rates, poorer responses to treatment, increased secondary, and/or contralateral cancer rates and worse overall mortality [1, 2]. Intentional weight loss has been associated with a decreased risk of breast cancer specific mortality in breast cancer survivors suggesting a potential cancer risk‐reduction benefit [1, 2, 4]. Therefore, we conducted a pilot study to determine the feasibility of a 20-week telehealth group-based weight loss intervention for management of obesity, the **G**roup-bas**E**d **T**elehealth behavioral **WE**ight **L**oss (GET-WEL) Program [3], which was based on the validated Diabetes Prevention Program [5]. The GET-WEL Program was led by a behavioral weight loss trained psychologist who implemented virtual sessions focused on nutrition, exercise, stress, emotion management and lifestyle modification strategies. Participants were encouraged to attend weekly sessions that were offered at two times, allowing flexibility in choosing which session suited their schedule. Participants were provided with a digital scale to track their weight progress with access to MyFitnessPal.com for personal fitness and nutrition tracking. Twenty-one women enrolled in the pilot program [15 (71%) White, 5 (24%) Black, and 1 Asian (5%)]. The results demonstrated lower weight loss efficacy in Black participants who on average ± SD lost 1.9% ± 6.6% of their baseline body weight compared to 7.5% ± 3.8% in White participants (*p* = 0.03) [3].

We subsequently conducted a qualitative study of GET-WEL participants and non-participants (i.e., participants invited but who declined participation) to identify barriers, facilitators, and perceptions of the telehealth weight loss intervention [6]. Among twenty-four total qualitative interviews, 9 were GET-WEL participants (8 White and 1 Black BCS) and 15 were GET-WEL non-participants (8 White, 6 Black, and 1 Asian BCS). Participants reported not enrolling in GET-WEL due to lack of knowledge that the program was enrolling, need for access to healthy foods within their community, and need for additional support to ease competing work/life priorities [6]. Limited research on understanding multilevel factors associated with weight loss barriers among Black breast cancer survivors cites both individual and neighborhood factors collectively influence weight loss; however, there is a paucity of literature focused on elucidating the granular factors and how to implement change in a weight loss intervention [7]. Given that our pilot GET-WEL study noted less overall weight loss among Black BCS, and our qualitative study listed some barriers to the enrollment of White and Black participants, our current study aims to utilize a community-based participatory research approach to assess barriers and facilitators of implementing a behavioral weight loss intervention in a predominantly Black community. 

## Methods

This study utilized principles of community-based participatory research (CBPR) to conduct qualitative semi-structured interviews. CBPR is a collaborative research framework which implements community-driven research approaches involving stakeholders, community members, and researchers to drive social change [8]. Utilizing principles of CBPR which mandate community access via endorsement from a trusted community leader with constituents, we partnered with the Director of Community Health Education Services for Chester City, PA [8]. Chester City, PA was chosen because it is a city in Pennsylvania with a high breast cancer age-adjusted incidence rate of 136 per 100,000 females as reported between 2017 and 2021 by the Pennsylvania Department of Health [9]. In addition, the population is predominately Black at 71.7% and has a significant obesity rate at 45%, and the U.S. Census recorded a poverty rate of 30.8% that is significantly greater than the national average of 11.5% [10]. Therefore, successful implementation of the GET-WEL program would address an unmet need to reduce obesity and breast cancer risk in this at-risk community and potentially serve as a model for implementation in other at-risk communities. Eight BBCS approached a member of the research team to participate and were subsequently enrolled in our study at a Chester Community Engagement event for Breast Cancer Awareness in Chester City, PA.

Inclusion criteria included English-speaking, non-Hispanic Black females greater than 18 years of age, interested in a weight loss intervention and currently residing in Chester City, Pennsylvania. Participation was voluntary; participants had the option to opt out at any time and no compensation was provided for qualitative interviews.

Prospective interviewees were identified as eligible by the study team at a Chester Community Engagement event for Breast Cancer Awareness in Chester City, PA. Eight BBCS approached a member of the research team at the event and were contacted subsequently by phone call and/or text messaging, based on participant preference. All 8 women were subsequently enrolled and participated in our study. Interview questions were influenced and designed using *The Health Belief Model and Preventive Health Behavior Model*, which provide a validated conceptual framework that focuses on understanding which factors influence individual health decision making in order to better tailor health interventions and lead to more effective health promoting behaviors, including health threats, barriers, and self-efficacy [11]. The interview questions integrated themes from this model such as barriers to exercise and understanding obesity. Interviews were conducted between October and November of 2024 using a semi-structured interview guide focused on perceptions, facilitators, and barriers to weight (Appendix 1).

Deidentified demographic questions were obtained prior to the interviews via a short survey which was collected and coded. The demographic questions comprise age, racial identity, marital status, and employment status. Of the eight participants, the ages ranged from 35 to 75 years; all were female BBCS. Regarding marital status, 3 identified as currently married, 3 divorced, 1 single, and 1 widowed (Table 1). All noted an interest in weight loss interventions.

**Table 1:** Socio-demographics and participant characteristics

Variable	*N* or as noted
Sample size	8
Mean age range (years)	55–64
Female	8
Black race	8
Marital status	
Single	1
Married	3
Divorced	3
Widowed	1
Education	
Less than high school	1
Completed high school or GED	2
Completed college	4
Masters/doctorate	1
Employment	
Unemployed	0
Part-time employment	2
Full-time employment	4
Retired	2

The interviews were 45 min in duration, not including the 15 min pre-interview demographic questionnaire which was not audio recorded. Interviews were voice recorded and professionally transcribed verbatim. All information was deidentified prior to transcription. The quality of the transcriptions was confirmed by members of the study team. The information was subsequently coded and analyzed by two members of the study team (EDJ and SW). The analysis utilized an integrated approach including mixed methods research principles for coding and thematic analysis which is an iterative process of determining theories of themes and patterns that are present in the data. The two coders independently coded 100% of the transcripts, meeting to resolve inter-coder differences and ensuring reliability. The framework, codes, and thematic analysis were categorized utilizing NVivo software. After coding, thematic reports were reviewed and compared for consistency. Thematic saturation was achieved based on the criteria of redundancy, with repetitive themes derived from the data of the 8 participants. Examples of responses are included within each theme. Information was subsequently coded and analyzed via comparative thematic analysis [12]. Research was determined to be exempt by the University of Pennsylvania Internal Review Board (IRB) and conducted in accordance with the ethical standards (IRB #857096). Each participant received an overview of the study and provided verbal consent prior to enrollment.

## Results

### Themes

Themes are presented in order of discussion and list an example quote from participants for each theme.

#### Theme 1: Perception of community nutrition and physical education

When participants were asked about their current perception of nutrition and physical activity in their community, they emphasized their belief that education about nutrition will have an impact on their ability to manage their weight and health. Many participants felt that the etiology of weight gain originated from early lack of education about nutrition, particularly originating with youth in the community and the development of life-long habits.

*“Once they learn what to eat and how to eat, I think it'll make a big difference.”*

#### Theme 2: Constant visibility and integration in the community

Participants described the need for ensuring programs with resources for the community are widely disseminated throughout to improve visibility. This was made clear when a portion of the discussion focused on exchanging information about community-based programs and access to resources with one another. Some participants were aware of certain resources while others were not. Participants also emphasized the need for sustainability, emphasizing the need for resources to persist in the community.

*There’s a need for people to know what programs are being offered and how they can become part of those programs”*

#### Theme 3: Healthy eating barriers

When discussing perceptions of nutrition and physical barriers that community members face, participants reported the importance of financial, environmental, and time barriers and access. Some participants focused on the financial restrictions of incorporating healthy food options into their diet while others focused on the difficulty with eating well and having access to healthy options before, during, and after long work hours.

Regarding incorporating seafood into a balanced diet, one participant stated *“I used to buy them, but they’re so expensive now”.*

*“I think you have to think about the economy too. You have people who may not wanna be unhealthy, but based on their situation, environment, financial situation, um, their economics, they can’t”*

Another participant noted the difficulty in eating a balanced healthy meal each day due to limitations in her work schedule, *“So I’m really only getting like one meal in a day because I’m working.”*

#### Theme 4: Physical activity barriers

Physical activity barriers in the community included financial barriers such as expensive gym memberships and the need to make those more financially accessible. The alternative for most participants includes walking, for which a consensus was reached about its amenability to all ages, activity levels, access, and financial statuses; however, the community has public safety concerns which pose a significant barrier.

*“It would be to our advantage to get into programs that are free.”*

*“I used to go for walks years ago but now you don’t feel safe because of the crime.”*

#### Theme 5: Perceptions of technology incorporation

When participants were prompted about their use of technology and their perceptions about its incorporation to facilitate healthy lifestyles, there was some initial resistance related to the learning curve required to implement technology into everyday lives. There was also mention of feeling as if they were not picking up on the use of technology as quickly as they would have liked, oftentimes feeling judged or criticized, which led to quick discontinuation.

*“Everybody don’t know how to do that. Only the young people know how to do that.”*

*“I'm not learning it quickly.”*

*“We do need training. I have to do things at least two or three thing,-- two or three times in order for me to understand or, you know, to understand what I'm doing.”*

However, some participants did note that they utilize smart watches or pedometers on their telephone and find them relatively easy to use. Some participants also cited financial barriers with updating devices, apps, and models.

#### Theme 6: Peer influence

An emphasis on the positive reinforcement that a group model offers to an intervention program both among participants as well as influencing additional community members to incorporate healthy habits.

*“So, you have to be a model to influence other people--Then once you start, maybe they’ll join on with you.”*

### Recommendation for ideal program

When prompted about ideal program recommendations, participants reported the need for this ideal program to be inexpensive and integrated into community-based facilities, as well as the need for diverse exercise/course offerings which included aerobics or yoga. Participants voiced a strong emphasis or desire for nutritional support such as cooking classes and examples of healthy food options to generate engagement and excitement. Also highlighted was the critical importance of a hybrid schedule offering flexibility to incorporate sessions into daily routine, particularly among many of the participants with busy employment and home lives.

*“Need for an affordable fitness center”*

*“More aerobics, yoga or dance”*

*“What about a cooking class?”*

*“Nutritionist -focused on creating healthy meals, like grilled chicken or how to make vegetables soups.”*

*“Available for me. And, you know, I can’t make it at 4 o’clock, but I can make it at 7. Or I can even make it later when, uh, when everything is done.”*

## Discussion

Recent studies focused on BBCS and behavioral weight loss as an intervention to reduce cancer mortality have cited mild to moderate weight loss with weight gain post intervention [3, 13–15]. Although weight loss intervention programs are currently being studied and are gaining traction, such as the Breast Cancer WEight Loss (BWEL) trial, an ongoing randomized, controlled trial designed to determine whether weight loss after breast cancer diagnosis can reduce the risk of cancer recurrence in women with overweight or obesity, there remains a paucity of literature focused on specific barriers and facilitators needed to improve weight loss among BBCS [16]. We demonstrated in our pilot GET WEL study that BBCS were less likely to enroll and only 20% of Black participants lost a clinically significant amount of weight as compared to White participants; therefore, we focused on understanding these barriers [3]. The most prominent barriers highlighted in our groups included the focus on a multimodal approach including nutrition education, encouraging physical activity by reducing financial barriers and use of basic technology.

When considering nutrition education, participants discussed providing healthy food options near the community such as access to farmers markets and education of lower cost healthy alternatives. Encouraging physical activity by reducing financial barriers included identifying subsidized gym memberships. Walking as a form of activity for weight loss was the general consensus; however, important additional barriers evolved including safety concerns with walking in the neighborhood. Participants discussed developing group- or peer-based programs to utilize local safe walking paths which both ameliorate safety concerns and offer a cost-effective alternative to gyms. Given that walking was considered a common form of exercise, we discussed tracking its effectiveness for weight loss. This yielded hesitation when discussing technology-based platforms and devices, such as activity trackers for weight management. Participants expressed interest in courses aimed at lowering the learning curve with these applications. They reiterated that they were eager to learn but noted they require more time to understand how to use new technology and expressed the need to learn in an environment free of judgement.

Importantly, our groups concluded with focusing on the Design, Recruitment, Implementation and Sustainability of ideal weight loss programs. In regard to *design*, participants focused on hybrid exercise and nutrition classes. They requested exercise programs with diverse exercise offerings. Participants were interested in various physical activities including yoga and tai chi. Community members strongly emphasized the need for nutritional support with integration of nutritional education. Cooking classes were recommended as a method to easily integrate nutrition into daily meals. The importance of a hybrid schedule comprising both in-person and virtual class options was recommended. Offering class time flexibility, accessibility and improved access to sessions in the community was viewed as particularly important given community members felt increased daily living demands can be inflexible. Preference for a prerecorded video of an exercise class allowed for easy daily integration. When discussing *recruitment and implementation*, participants reiterated connecting with current community health advocates. They discussed partnering with these trusted local leaders and prominent figures of the community to build visibility and trust. Implementation of this weight loss program with active community health coaches would improve engagement. Finally, *sustainability* in the community was emphasized by focusing on integrating in current community spaces such as churches and community centers to reduce costs and maintain continuity. The design, recruitment, implementation and sustainability factors highlighted by community members for this ideal program could effectively be implemented into our upcoming iteration of GET-WEL to improve its implementation among Black breast cancer survivors (BBCS).

Importantly, there were several limitations of this study. The study comprised a small sample size of eight participants. In addition, all interviewees were recruited from a Breast Cancer Awareness Health event in Chester City, Pennsylvania, which may introduce self-selection bias and may affect the generalizability of the study. Participants were not asked to validate their weight or BMI with a scale; therefore, some participants may not meet the clinical criteria of overweight or obesity. Specifying BMI as an inclusion criterion may have limited our sample size further as many participants also noted that they were unaware if they were considered to have overweight vs obesity by standard clinical metrics. Future studies should focus on increased sample size and confirm individual BMI with clinical metrics. In addition, for the purposes of this study and resource limitations, we only included English speakers, which inherently limits diversity in the participant population; however we feel it is important to also make this intervention accessible for non-English speakers as well. In addition, responses from the participants may lean toward more exaggerated, positive comments, as they may be subjected to the Hawthorne effect when in a group setting [17]. Regardless of the following limitations, strengths of this study include our ability to delineate important barriers to equitable implementation of our weight loss program among BBCS utilizing principles of Community-Based Participatory Research by leveraging access to the constituents of the Director of Community Health Education Services for Chester, PA, a trusted official within the community [8].

Focusing on community-based principles to understand the granular barriers associated with weight loss among Black breast cancer survivors is necessary given the need for effective implementation of intentional weight loss among this population. Our results will help inform the design, recruitment, implementation, sustainability, and scaling of future inclusive weight loss programs, such as future iterations of GET-WEL by incorporating factors that improve diversity and efficacy among Black breast cancer survivors.

## Data Availability

No datasets were generated or analyzed during the current study.

## Appendix 1

### Interview questionnaire: perceptions, facilitators and barriers

Perceptions

How common is obesity in your community?

1. What do you think are the health consequences of obesity?
2. What do you think we can do about obesity in your community?
3. Do you find it difficult to be healthy? Do you think others in the community find it difficult to be healthy?
  - If so, what makes it difficult to be healthy?
  - If not, what makes it easy to be healthy?

Facilitators

4. What strategies could work to help people in your community improve their diet?
5. What strategies could actually work to help people in your community increase their physical activity?
6. What kinds of things could people do personally to help their family members become more physically active?
7. What things could people do personally to help their family improve their diet?
8. How can technology help people lose weight?
  - Smartphone apps
  - Exercise equipment such as exercise bikes, treadmills etc.
  - Virtual conferences with Nutritionist, psychologist etc.
9. What are the most helpful interventions that PENN medicine or other organizations can do to help tackle obesity in your community?

Barriers

10. What are the challenges that people in your community face to stay healthy?
11. What do you think about the following other barriers?
12. Why?
  - Financial barriers?
  - Multiple demands?
  - Personal motivation?
  - Limited healthy food options?
  - Healthy food taste?
  - Lack of access to places where healthy lifestyles are easier?
    i. Grocery Stores
    ii. Parks
    iii. Gyms
  - Neighborhood conditions?
  - Technology barriers?
  - Weather?

What does your ideal healthy intervention look like?

## Appendix 2

### Themes, codes and participant quotes

**Table:**

Themes	Codes	Quotes
Perception of Community Nutrition and Physical Education	Community education regarding obesity and nutrition Community options to optimize health Community gym resources for the youth	*"Once they learn what to eat and how to eat, I think it’ll make a big difference.“* *"They are eating the wrong foods"* *“I think you have to think about the economy too. You have people who may not wanna be unhealthy, but based on their situation, environment, financial situation, um, their economics, they can’t”* * “I don’t know of gym resources for kids here”*
Community Visibility	Constant visibility and integration in the community	*“There’s a need for people to know what programs that are being offered and how they can become part of those programs”* *"If you want to be successful in your neighborhood, I think you should be part of your neighborhood."*
Healthy Eating Barriers and Perceived Restrictions	Financial barriers affecting food consumption choices Difficulty making healthy food choices while out	*“I used to buy them (seafood), but they’re so expensive now”* *“So I’m really only getting like one meal in a day because I’m working.”*
Physical Activity Incorporation Barriers	Financial barriers of gym membership Crime affecting safety Availability	*"It would be to our advantage to get into programs that are free.“* *“I used to go for walks years ago but now you don’t feel safe because of the crime.”* * “Saturday would be the best day, most people don’t work or go to church on Saturday”*
Perceptions of Technology Incorporation	Financial barriers of smart technology for weight loss Learning curve utilizing technology	*“It’s expensive, then there are the constant updates…”* *“Everybody don’t know how to do that. Only the young people know how to do that.”* *“I’m not learning, learning it quickly”* *“We do need training. I have to do things at least two or three thing,– two or three times in order for me to understand or, you know, to understand what I’m doing”*
Comradery Advantages	Leading by example Comradery assists with exercise maintenance	*“They notice everything that you do and that you exercise”* *"So you have to be a model to influence other people–Then once you start, maybe they’ll join on with you.”*
Recommendation for Ideal Program	Ideal facilities need to be inexpensive Diverse exercise offerings Emphasis on nutritional support Need to learn how to cook healthy and tasty meals Hybrid schedule offers flexibility to incorporate sessions into daily routine	*“Need for an affordable fitness center”* *“More aerobics, yoga or dance”* * “What about a cooking class?”* *“Nutritionist focused on creating healthy meals, like grilled chicken or how to make vegetables soups.”* *“Available for me. And, you know, I can’t make it at 4 o’clock, but I can make it at 7. Or I can even make it later when, uh, when everything is done”*
